# Impact of a New Reimbursement Program on Hepatitis B Antiviral Medication Cost and Utilization in Beijing, China

**DOI:** 10.1371/journal.pone.0109652

**Published:** 2014-10-16

**Authors:** Qian Qiu, Yan Li, Xiao-wan Duan, Li-kun Yang, Yu Chen, Hui Li, Li Wang, Zhong-ping Duan

**Affiliations:** 1 Dept of Epidemiology and Biostatistics, Institute of Basic Medical Sciences Chinese Academy of Medical Sciences, School of Basic Medicine Peking Union Medical College, Beijing, China; 2 Liver center, Beijing You'an Hospital of Capital Medical University, Beijing, China; MOE Key Laboratory of Environment and Health, School of Public Health, Tongji Medical College, Huazhong University of Science and Technology, China

## Abstract

**Background:**

Hepatitis B virus (HBV) infection is a significant clinical and financial burden for chronic hepatitis B (CHB) patients. In Beijing, China, partial reimbursement on antiviral agents was first implemented for the treatment of CHB patients in July 1, 2011.

**Aims:**

In this study, we describe the medical cost and utilization rates of antiviral therapy for CHB patients to explore the impact of the new partial reimbursement policy on the medical care cost, the composition, and antivirals utilization.

**Methods:**

Clinical and claims data of a retrospective cohort of 92,776 outpatients and 2,774 inpatients with non-cirrhotic CHB were retrieved and analyzed from You'an Hospital, Beijing between February 14, 2008 and December 31, 2012. The propensity score matching was used to adjust factors associated with the annual total cost, including age, gender, medical insurance type and treatment indicator.

**Results:**

Compared to patients who paid out-of-pocket, medical cost, especially antiviral costs increased greater among patients with medical insurance after July 1, 2011, the start date of reimbursement policy. Outpatients with medical insurance had 16% more antiviral utilization; usage increased 3% among those who paid out-of-pocket after the new partial reimbursement policy was implemented.

**Conclusions:**

Direct medical costs and antiviral utilization rates of CHB patients with medical insurance were higher than those from paid out-of-pocket payments, even after adjusting for inflation and other factors. Thus, a new partial reimbursement program may positively optimize the cost and standardization of antiviral treatment.

## Introduction

Hepatitis B viral (HBV) infection is a global public health problem. A universal vaccination program against HBV has been carried out in China since 1992, contributing significantly to reduced HBV surface antigen (HBsAg)-positive rate (9.75% in 1992 to 7.18% in 2006) [1]. However there were still estimated 20 million individuals with HBV in China [2], representing a financial burden to themselves and their families.

Individuals with chronic HBV have a 15–40% probability for developing compensated cirrhosis, decompensated cirrhosis and hepatocellular carcinoma, which significantly affect patient treatment outcomes and quality of life measures. Also, healthcare costs can increase with disease progression [3], [4]. Thus prevention or delay of hepatic HBV progression may offer better outcomes and significant savings.

At this time, antiviral treatment can reduce fibrosis progression and cirrhosis and prevent further disease progression, including hepatocellular carcinoma [5], [6], [7]. Over the past 10 years, considerable improvement in HBV treatment has occurred, including development of antivirals such as interferon (IFN)-based therapy and nucleos(t)ide analogs (NAs). Four NAs: lamivudine (LAM), adefovir (ADV), entecavir (ETV) and telbivudine (LdT) have been approved in China. The costs and utilization of these drugs in China have yet to be described, especially in the setting of a new reimbursement program in Beijing.

In Beijing, no reimbursement was available for HBV antivirals prior to July 1, 2011: all drug expenses were paid by the individual patients. This may have resulted in low compliance due to cost [8]. At this time, IFN and NAs are listed with the National Reimbursement Catalog of Drugs for Basic Medical Insurance and partial reimbursement has been available since July 1, 2011 in Beijing, mainly designed for civil residents. Patients could receive a 75–85% reimbursement of the cost between a deductible of 1,800 yuan and a ceiling of 20,000 yuan. This should increase affordability and patient compliance but data have not been collected and studied. Thus, we investigated this new partial reimbursement program on cost, patient demographics, and antiviral utilization.

With inpatient and outpatient electronic data from Beijing You'an Hospital, a university affiliated infectious disease hospital in Beijing, China, we estimated average annual treatment costs and the proportion of costs of drugs (including antivirals), laboratory tests, and related fees to describe the current treatment patterns for CHB patients to understand how the new partial reimbursement policy affects healthcare costs, patients affected and utilization of antiviral drugs.

## Materials and Methods

### Ethics statement

The study protocol was approved by the Ethical Review Committee of Beijing You'an Hospital, Capital Medical University and Institute of Basic Medical Sciences Chinese Academy of Medical Sciences. The team obtained informed consent from hospital authorities for data collection. No informed consent, written or oral, was obtained from the participants because only the electronic medical records were retrieved to do the analysis and only necessary data for study objectives were extracted. Deidentification was done to assure confidentiality of the study data.

### Study population

A retrospective cohort study was conducted on CHB patients at You'an Hospital, Beijing, between February 14, 2008 and December 31, 2012. Patients were diagnosed by the “Asian-Pacific consensus statement on the management of CHB [9]”. Patients co-infected with hepatitis A, C, D and E, human immunodeficiency virus, cytomegalovirus or those admitted to the hospital due to diagnosed liver cirrhosis or hepatocellular carcinoma or other diseases or conditions, including pregnancy, glomerulonephritis, uremia, complications of metabolic syndrome, tumors, and cardiovascular diseases, were excluded. Also, non-residents of Beijing were excluded.

### Data collection

Demographic characteristics, clinical information and medical expenses for each patient visit were retrieved from electronic medical records. Demographic characteristics included, gender, birth date, admission (or visit) date, inpatient discharge date, and health insurance type were documented. Health insurance type was categorized into 3 groups: out-of-pocket, medical insurance and other kinds of insurance that included the new rural cooperative medical scheme (NCMS), free medical service, commercial insurance for medical care which was voluntary paid by patients themselves and compensated by insurance companies, and so on. Baseline and laboratory tests were also retrieved from the electronic medical record to identify treatment indicator. These tests included routine biochemical tests (serum alanine aminotransferase), virological markers (serum HBV DNA) and hepatitis B e antigen (HBeAg) status. Direct medical expenses included any expenditure from registration, lab tests, imaging tests, medications, hospital stays and hospital supplies. Indirect cost, including medical leave, time off, and productivity loss, was not collected. We also collected detailed antiviral utilization, including drug names, dosage, costs, and treatment durations. Indirect costs, reflecting the value of lost productivity but other losses, such as patient travel and medical assistance expenditures, however, were not included.

### Cost estimation

All costs were inflated to Dollars in the year 2012 using the Consumer Price Index for Health in Beijing [10] and also expressed in US dollars in the year of 2012. The exchange rate of RMB to USD was 6.3 to 1. Annual inpatient and outpatient costs for each patient were calculated according to the following formula:
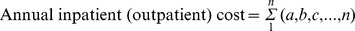



Where a, b, c…, z represents actual costs occurred for each visit/hospital admission of that year, such as drug costs, lab and imaging tests, registration, and all related costs. Finally, n represents visit times for each patient each year.

Because the electronic medical record system in You'an Hospital was established on February 14, 2008, records prior to this date could not be collected, which may lead to an artificially lower annual cost per person. Thus, the average proportion of visits before February 14 from 2009 to 2012 was used to adjust the visit time and the corresponding annual cost per person in 2008.

To explore the effect of the new partial reimbursement program on care costs, patient composition, and antiviral utilization, we divided 2011 data into two parts: costs in the first and second half of the year. Each half-year cost was adjusted by the corresponding half-year cost in 2010 and 2012 using the following formula:
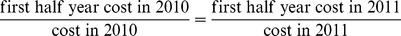


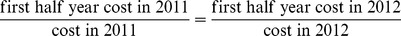



### Propensity score matching

The treatment cost was greatly influenced by the socio-economic status, medical technology, the health care system, the patients' disease severity and healthcare awareness [8]. Logistic regression was adopted to estimate the propensity score (PS). The dependent variable was admission year and the independent variable (covariates) were age, gender, medical insurance type and treatment indicator (patients were considered treatment indicated if met one of the following conditions: (1) serum alanine aminotransferase over two times upper limit of normal (40 U/L); (2) log_10_ DNA> = 5 copies/ml for HBeAg (+) patients; (3) log_10_ DNA> = 4 copies/ml for HBeAg (−) patients [11]). All covariates were entered into the logistic regression model and the probability of being in the case group (known as PS) was then calculated for each patient. As a 1∶1 matching was adopted thus outpatients in 2008 were considered as the control group due to the smallest sample size. Outpatients in 2009, 2010, 2011 (first half-year), 2011 (second half-year) and 2012, were matched as the case group to the control group by PS within a range of 0.1 standard deviation respectively. Then, data from 2008 that had been matched to all five years and corresponding pairs were selected so that baseline characteristics would be comparable among the six time periods. For inpatients, the smallest sample size was from the first half of 2011. So, this was used as the control group.

### Statistical analysis

The average annual cost for inpatients and outpatients was expressed as median (first quartile and third quartile). The proportion of the cost of laboratory tests, medications (especially antivirals) was calculated by the annual total cost of laboratory tests and medications divided by the total costs for all patients.

Chi-square test was used to detect differences in the proportion of antiviral therapy among outpatients with different medical insurance types. SAS 9.2 (Windows, SAS Institute Inc., Cary, NC) was used to perform the statistical analysis.

## Results

**Figure 1:**
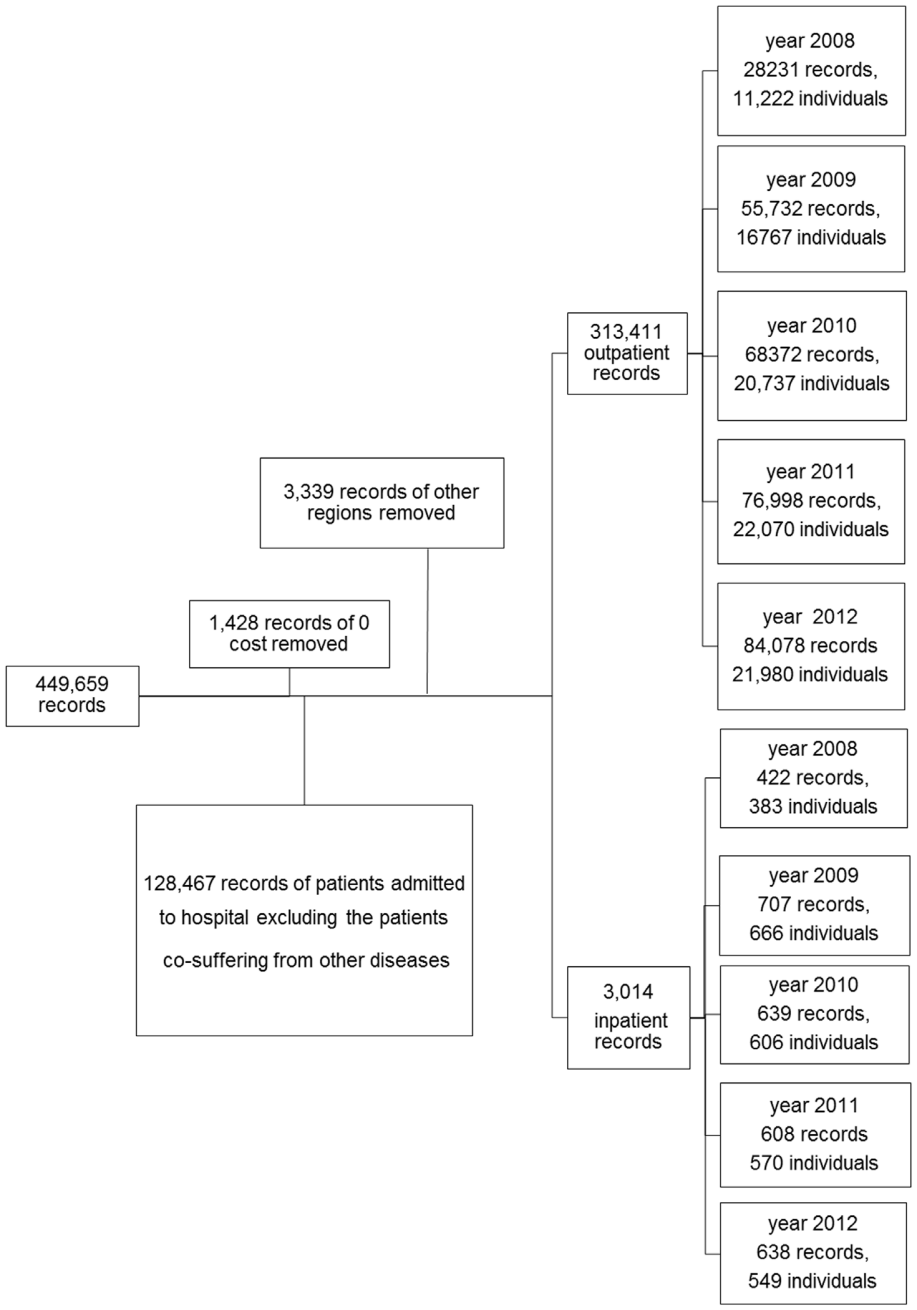
Patient selection procedure.

A total of 444,900 outpatients and 4,759 inpatients medical records of CHB patients were documented from February 14, 2008 to December 31, 2012. A total of 1,428 medical records with no treatment, 128,467 cases admitted to the hospital with other diseases or conditions, as well as 3,339 cases from outside of Beijing who were covered by different local reimbursement policies from those in Beijing were excluded, leaving 313,411 outpatients and 3,014 inpatients medical records. **Figure 1** depicts the selection procedure.

### Subject characteristics

Patient demographics among different admission years are depicted in **Table 1**. Age, gender, health insurance type and treatment indicator was significantly different among admission years. Patients in each admission year were predominantly male and the mostly male was in 2008 for outpatients and 2009 for inpatients. The proportion of patients who paid out-of-pocket decreased and those with medical insurance increased annually. To allow a common ground for comparison among different admission year, PS matching were conducted by selected key characteristics. PS matching resulted in a matched sample size of 10,587 for outpatients and 186 for inpatients for each admission year and the distribution of key confounders, including age, gender, insurance status and treatment indicator, was similar in outpatients and inpatients in different admission years (**Table 2**).

**Table 1:** Demographic characteristic of CHB patients from 2008 to 2012.

			Age(year)		Gender		Insurance type (%)				Treatment indicated	
	Year	N	Mean±Sd	*p*	Male%	*p*	Out-of-	Medical	Else	*p*	%	*P*
							pocket	insurance				
Outpatient	2008	11222	39.0±13.7	<0.0001	68.6	<0.0001	68.3	23.8	7.9	<0.0001	12.8	<0.0001
	2009	16767	38.2±13.5		67.5		67.1	22.8	10.1		22.8	
	2010	20737	38.1±12.9		65.5		61.0	26.3	12.7		23.9	
	2011	22070	39.2±12.3		63.4		52.6	36.6	10.8		20.3	
	2012	21980	39.4±12.8		62.8		49.1	42.7	8.2		20.0	
Inpatient	2008	383	34.5±12.1	<0.0001	71.0	<0.0001	67.6	25.6	6.8	<0.0001	36.0	<0.0001
	2009	666	35.7±12.4		77.3		67.8	23.9	8.3		37.8	
	2010	606	36.1±12.9		74.6		58.1	21.9	20.0		40.1	
	2011	570	41.7±13.6		71.4		43.3	36.1	20.6		47.0	
	2012	549	41.1±12.8		69.0		27.0	53.9	19.1		42.1	

**Table 2:** Demographic characteristic of CHB patients from 2008 to 2012 after PS matching.

			Age(year)		Gender		Insurance type (%)				Treatment indicated	
	Year	N	Mean±Sd	*p*	Male%	*p*	Out-of-	Medical	Else	*p*	%	*P*
							pocket	insurance				
Outpatient	2008	10587	39.1±13.0	0.8306	67.9	0.4289	66.4	25.2	8.4	0.7495	13.5	0.7192
	2009	10587	39.0±12.8		68.7		67.3	24.3	8.4		13.5	
	2010	10587	39.0±12.7		68.0		66.6	25.1	8.3		13.5	
	First half of 2011	10587	39.0±12.3		67.7		66.8	25.1	8.1		14.0	
	Second half of 2011	10587	39.2±12.8		67.5		66.6	25.2	8.2		13.4	
	2012	10587	39.4±12.6		68.4		67.0	25.2	7.8		13.6	
Inpatient	2008	186	39.0±11.5	0.5171	74.7	0.682	61.8	26.4	11.8	0.0244	41.4	0.1364
	2009	186	39.0±12.5		77.4		53.2	37.1	9.7		43.0	
	2010	186	38.5±12.6		76.9		53.2	28.5	18.3		43.0	
	First half of 2011	186	38.5±12.0		72.6		55.9	33.9	10.2		44.1	
	Second half of 2011	186	41.2±12.1		73.1		47.3	38.2	14.5		47.9	
	2012	186	40.8±12.3		71.0		46.8	37.1	16.1		50.0	

### Cost analysis

The direct costs and cost composition from year 2008–2012 after PS matching were shown in **Table 3**. Two apparent inflection points may have explained the outpatients cost changes in 2009 and the second half of 2011. For inpatients, the annual average costs were higher than outpatients in the same year and annual costs for inpatients increased yearly, peaking in the second half of 2011.

**Table 3:** Annual total cost and the composition among CHB patients from 2008 to 2012.

	Year	Total	Antiviral cost (%)	Medicine without antivirals (%)	Test (%)	Others (%)
		Median(p25,p75)				
Outpatient	2008	179.2 (67.0,481.5)	53.1	32.3	10.9	3.7
	2009	219.9 (74.4,709.1)	57.7	28.2	10.8	3.3
	2010	201.1 (66.7,689.6)	56.4	27.0	12.8	3.8
	First half of 2011	170.1 (66.9,483.1)	56.8	23.4	15.8	4.0
	Second half of 2011	219.5 (82.0,605.1)	63.0	19.6	14.1	3.3
	2012	217.1 (77.0,729.1)	63.9	17.3	15.4	3.4
Inpatient	2008	1274.8 (529.5,2409.8)	8.3	52.3	20.6	18.8
	2009	1590.7 (614.0,2854.8)	11.5	49.8	20.6	18.1
	2010	1612.2 (679.6,2491.0)	12.5	49.4	20.7	17.4
	First half of 2011	1357.4 (299.2,2851.1)	9.2	52.7	14.1	24.0
	Second half of 2011	2060.1 (641.7,3623.6)	9.5	54.7	14.4	21.4
	2012	1697.9 (496.8,3616.4)	13.2	48.8	13.6	24.4

Composition analysis revealed that the proportion of drug costs nearly identical (∼80%) for outpatients and 60% for inpatients during the study period. After a steady growth prior to July 1, 2011, the implementation of the partial reimbursement policy, antiviral costs jumped from 53 to 64% for outpatients and from 8 to 13% for inpatients. The proportion of laboratory costs increased from 11 to 15% for outpatients but decreased from 21 to 14% for inpatients.

### Anti-viral drug and medical service utilization

**Figure 2:**
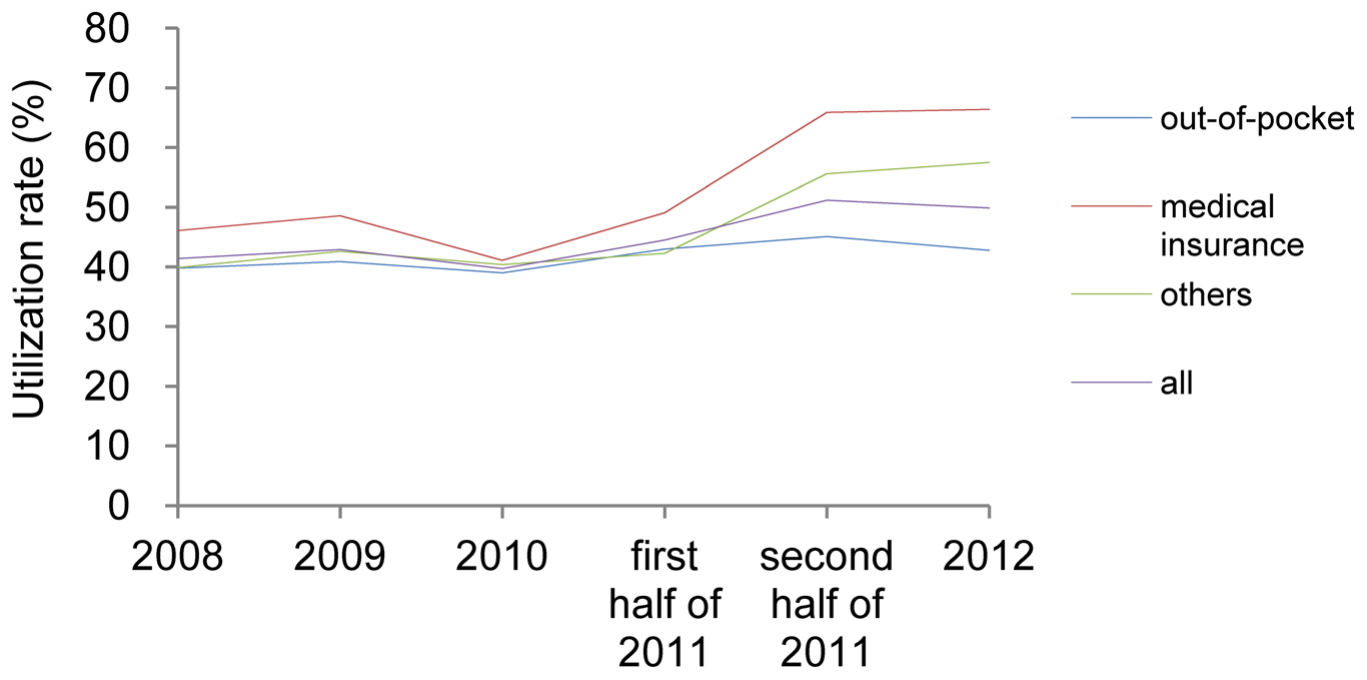
Antiviral utilization among CHB patients with different insurance types from 2008–2012.

Because inpatients focused on symptomatic treatment, we only analyzed antiviral drug utilization for outpatients. We found that the antiviral utilization had grown rapidly since the second half year of 2011 (**Figure 2**). The antiviral utilization was around 40% before July 1, 2011 and jumped to 50% after then.

**Figure 3:**
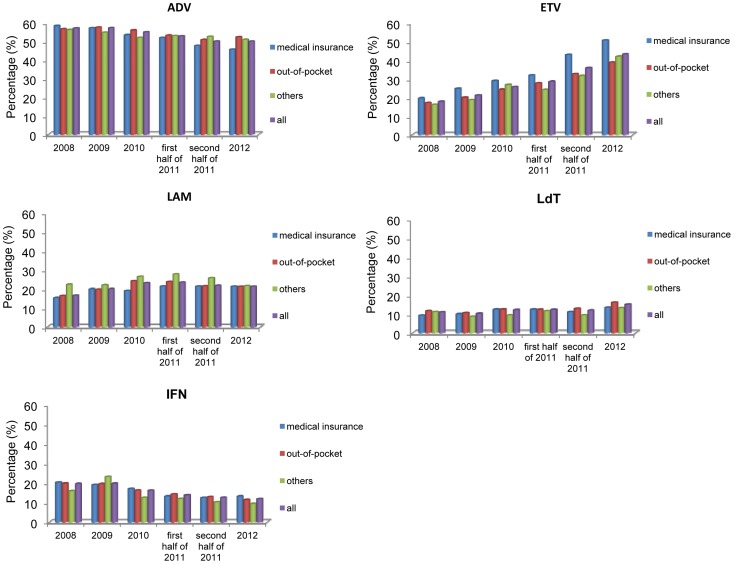
Antiviral composition of outpatients prescribed antivirals with different insurance types from 2008–2012. *Note: some patients treated with more than one antiviral at the same time may increase the sum of antiviral composition to more than 1 in each year.

Among outpatients who received antiviral treatment, ADV was the most frequently prescribed antiviral, comprising 50% of those prescribed during the 5-year period, even though utilization of this compound decreased slightly. IFN usage decrease from 20 to 12% after the new policy was implemented. However the proportion of patients using ETV increased 4% before July 1, 2011 and increased 10% after that time. LdT and LAM did not change much regarding usage (**Figure 3**).

### Effect of partial reimbursement policy on patients with different insurance

The new partial reimbursement program was only available to patients with medical insurance, so we divided patients into three insurance groups (out-of-pocket, medical insurance, and other types of insurance) to investigate the effect of the new partial reimbursement policy.

#### Total cost

Data indicate that the total annual cost was higher among both outpatients and inpatients with medical insurance compared to those who paid out-of-pocket, especially since the second half year of 2011. The total cost for outpatients with medical insurance increased 50% after the second half of 2011, compared to before. For patients who paid out-of-pocket, rates increased only 19% (Table 4).

**Table 4:** Annual total cost and the composition among CHB patients with different insurance types from 2008 to 2012.

		Total			Antivirals (%)			Medicine without antivirals (%)			Lab test (%)		
		Median (p25,p75)											
		Out-of-	Medical	Others	Out-of-	Medical	Others	Out-of-	Medical	Others	Out-of-	Medical	Others
		pocket	insurance		pocket	insurance		pocket	insurance		pocket	insurance	
Out-patient	2008	153.9 (60.2,429.3)	255.4 (100.4,649.8)	178.3 (67.2,480.6)	55.7	48.8	50.6	29.6	37.1	34.0	11.0	10.7	11.4
	2009	183.8 (64.8,589.9)	359.2 (120.3,1040.4)	227.1 (79.9,705.3)	59.0	55.9	56.2	26.1	31.4	30.0	11.5	9.8	10.5
	2010	175.9 (60.7,599.1)	293.5 (91.8,960.0)	192.4 (69.7,598.1)	59.7	51.3	51.1	23.1	33.4	31.3	13.4	11.7	13.2
	First half of 2011	153.5 (61.2,460.1)	227.8 (90.9,564.7)	155.1 (66.0,380.8)	59.8	54.0	53.5	20.2	26.9	25.1	15.9	15.3	17.3
	Second half of 2011	182.6 (70.8,530.6)	338.5 (120.6,838.7)	219.3 (97.5,556.7)	63.7	65.2	60.3	15.8	20.1	20.5	16.6	11.8	15.3
	2012	176.7 (63.9,586.5)	407.1 (127.2,1111.1)	237.8 (82.8,730.6)	60.6	68.9	65.0	17.9	16.3	17.1	17.7	11.9	14.6
In-patient	2008	1175 (483.3,2074.3)	1453 (836.0,2928.0)	1336.8 (469.3,1867.6)	8.1	8.1	9.9	53.6	51.7	46.8	20.1	20.9	22.9

		Total			Antivirals (%)			Medicine without antivirals (%)			Lab test (%)		
		Median (p25,p75)											
		Out-of-	Medical	Others	Out-of-	Medical	Others	Out-of-	Medical	Others	Out-of-	Medical	Others
		pocket	insurance		pocket	insurance		pocket	insurance		pocket	insurance	
In-patient	2009	1152.6 (280.9,2671.9)	2134.3 (1342.8,2949.1)	2097.7 (925.8,2923.0)	17.8	5.5	7.1	45.7	54.0	51.1	19.0	22.1	22.2
	2010	1466.6 (557.8,2230.7)	2194.4 (1482.2,3415.5)	1323.3 (372.9,2254.0)	18.8	7.5	4.9	43.3	56.1	52	20.9	19.5	23.2
	First half of 2011	1270.9 (212.9,2796.8)	1660.7 (594.9,3338.5)	1128.9 (297.2,2751.1)	16.5	3.0	10.8	46.6	55.8	55.3	14.1	13.4	15.5
	Second half of 2011	1098.2 (237.8,2524.8)	2706.6 (1685.7,4903.3)	2117.8 (1242.6,3281.3)	10.3	9.1	9.3	48.0	56.1	60.6	16.6	13.7	13.2
	2012	1351.7 (231.4,2677.5)	1794.1 (700.7,5188.8)	2236.5 (1572.1,4006.9)	10.9	15.8	11.3	50.1	45.2	54.2	17.0	12.5	11.0

#### Cost composition

Among outpatients, the proportion of costs for lab tests among the three insurance groups increased somewhat during the study period; drug costs increased more rapidly among those with medical insurance than those who paid out-of-pocket after the second half of 2011. Non-antiviral drug costs were not different between the two groups. For inpatients, a yearly decrease in lab costs was observed among all three groups and drug costs rose annually among the three groups (Table 4).

#### Antiviral utilization

Before the first half of 2011, antiviral utilization fluctuated ∼2% for outpatients who paid out-out-pocket. This rate increased from 43.0 to 45.1% after the new policy was implemented (Figure 2). For patients with medical insurance, however, the difference between different admission years before July 1, 2011 did not exceed 8% and increased from 49.1 to 65.9% after that time. Among patients with other insurance types, most were covered by NCMS or had free medical services. Antiviral utilization ranged from 41.3–45.2% for those with free medical care and from 39–45% for those with NCMS from 2008–2011 and this increased from 41.2 to 51.9% and from 45.2 to 60.9% after July 1, 2011, respectively. These increases were lower than experienced by those with medical insurance but higher than the costs for those who paid out-of-pocket (data not shown).

The proportion of outpatients with medical insurance treated with ETV increased from 30% to more than 40% with an annual growth of 34% since July 1, 2011, higher than before July 1, 2011. Annual growth of no more than 20% was observed among those who paid out-of-pocket and this was not different to costs prior to July 1, 2011. Decreased use of ADV among patients with medical insurance during the second half of 2011 was 8.3% of the previous year, which was twice that of patients who paid out-of-pocket. The yearly utilization trend for the other three antivirals was not different between patients with medical insurance and those who paid out-of-pocket (Figure 3).

For outpatients with free medical care, ADV use decreased from 50.0 to 38.8% and ETV use increased from 36.8 to 51.0% after July 1, 2011. For those with NCMS, ADV use decreased during the study period and ETV use was 10% lower than the use observed by individuals with free medical care in the same year before July 1, 2011. After the implementation of the new policy, this use was still lower than that of outpatients who paid out-of-pocket. Over time, ETV utilization grew among those with NCMS compared to outpatients with other insurance types (data not shown). Medical service utilization was observed higher among those with medical insurance compared to those who paid out-out-pocket. Proportion of patients who visited hospital three or more than three times a year increased from 39.2% in 2008 to 55.9% in 2012 among insured patients during the study period, while increased slightly from 26.8% in 2008 to 36.4% in 2012 for those paid out-of-pocket (data not shown).

## Discussion

Approximately 20 million Chinese live with HBV, and antiviral therapy is one of the effective ways of improving their lives even though cost is a significant barrier [7]. Using electronic data from a university affiliated infection specialty hospital in Beijing, China, costs and antiviral utilization for 92,776 outpatients and 2,774 inpatients with HBV from 2008 to 2012 were analyzed. Outpatient costs were less than inpatients in Beijing, Guangzhou in 2007 [12] and Shandong in 2010. Economic and social issues may explain these cost differences as well as technological advancements in each area. Estimating a disposable personal income of $5,353 in Beijing in 2012, the annual total cost for CHB patients was estimated to be ∼5–40% of that income. Thus, annual costs for CHB patients were significant burdens for patients and their families. Creating a practical reimbursement policy was needed to reduce this financial burden and increase care affordability for all patients so that they may receive adequate and timely treatment to prevent or reduce disease progression and ultimately extend survival.

Antiviral treatment is a priority for CHB patients to reduce the risk of complications and delay HBV infection progression [13], [14], [15]. Here, we studied the yearly growth of antiviral utilization among outpatients, specifically in the second half of 2011 and 2012, when a new partial reimbursement policy was offered in China. We noted that antiviral use increased and these drugs represented a large proportion of all medication costs that increased annually as other drug costs decreased over the same time. Also, outpatients were more likely to choose antiviral treatment after the new reimbursement policy was offered. Inpatients, however, chose antivirals less often and other medication costs were significant. Perhaps inpatients required more drugs other than antivirals. After the partial reimbursement policy in Beijing, utilization of antivirals may increase due to greater affordability, a concept supported by the fact that antiviral utilization in the second half of 2011 and 2012 among CHB outpatients with medical insurance increased more than those who paid out-of-pocket. Liaw and colleagues arrived at a similar conclusion: they reported a lack of adequate reimbursement was correlated to lack of adherence to treatment guidelines [8].

In our study patients, almost 50% of outpatients were prescribed ADV which was cheaper compared to the other NAs approved in China. From 2008–2012, ADV utilization decreased and ETV utilization increased and this was more pronounced for people with medical insurance after the new partial reimbursement policy was implemented. Thus, the new policy may have increased the antiviral utilization by increasing the affordability as well as enabling the use of more expensive drugs such as ETV, which offers better therapeutic efficacy and fewer side effects and drug resistance [16], [17], [18]. Further analysis showed that for those with free medical care ADV was less used, and ETV was more popular, compared to patients with NCMS and medical insurance. The NCMS was established in rural areas with lower household income, likely poorer medical technology and lower new drug coverage rates, compared with urban patients. Thus, these rural individuals may have chosen less expensive and more common antivirals.

Our study also showed that outpatients with medical insurance had more medical visits, indicating that a reimbursement policy may standardize antiviral treatment patterns, improve patient outcomes finally for those with medical insurance compared to those who paid out-of-pocket.

There are several limitations in our study. First, our patients were recruited from Beijing You'an hospital, one of the two largest infectious and liver disease hospital in Beijing, they might not have been representative of the general CHB population in Beijing. But the demographic characteristic in our study population showed that male to female ratios were 2 to 4, and the mean of age range was 38–42 years, facts consistent with the age and gender distribution of HBV infection in other studies [19], [20], [21], [22], [23]. Second, real-world based electronic medical records from You'an Hospital over a 5-year period from 2008 to 2012 were used to evaluate the effect of reimbursement policy on medical care cost, as well as antiviral selection and utilization, which may result in the different stage of HBV infection for different admitting years. Although we used a PS to match the factors that would affect the treatment and cost, other observed confounding factors, such as income, education level, were not adjusted. Third, we estimated the cost for outpatients and inpatients separately for each admission year. But it can represent the annually cost for CHB patients because a less than 10% overlap were observed among outpatients and inpatients for each admission year. Finally, the direct cost and medical service frequency data may be underestimated because only electronic medical records were used. The direct costs of patient who visited other hospitals or pharmacies would not be included.

In conclusion, direct medical costs and antiviral utilization for CHB patients with medical insurance increased more than those who paid out-of-pocket annually, especially after the new partial reimbursement was put into lace and after adjusting for inflation and patients' baseline characteristics. Thus, the new partial reimbursement policy positively optimized the cost and standardization of antiviral treatment, offering improved patient outcomes.
